# Telemedicine and E-Health: An Innovative Challenge in Pediatric Care

**DOI:** 10.3390/ijerph20032091

**Published:** 2023-01-23

**Authors:** Gianvincenzo Zuccotti, Valeria Calcaterra

**Affiliations:** 1 Department of Biomedical and Clinical Science, University of Milano, 20157 Milano, Italy; 2 Pediatric Department, Buzzi Children’s Hospital, 20154 Milano, Italy; 3 Department of Internal Medicine and Therapeutics, University of Pavia, 27100 Pavia, Italy

Telemedicine represents the practice of medicine by remote means, via real-time two-way communication between the patient and the healthcare provider [1]. It includes all health care and health system components and activities that are conducted through telecommunications and information technology [1].

In recent years, telemedicine use has increased in the pediatric age [2]. It is part of the ever-growing use of communications technology in children’s health care being utilized in prevention, management of disease, home health care, remote monitoring, diagnostic therapeutic assistance, and individual assistance plans [3]. It is applicable in emergency medicine, long-term chronic care, remote medical imaging, and many other pediatric applications [4]. Technological systems also offer the opportunity to reduce hospital admissions and to anticipate discharge, with important psychological and social advantages for children. The implementation of active domiciliary monitoring and the introduction of a homecare hospital provides continuous assessment of critical medical status, and clinically relevant issues [3].

A special role of technological systems and e-health components must be considered in children with disability and medical fragility to support their multiple health needs [5], offering continuity and equity of healthcare access [1].

The technological approach can contribute to the reorganization of pediatric health care, supporting innovative systems centered on the person and offering increased access to health services, cost-effectiveness, enhanced preventive and educational initiatives, as well as improved health outcomes with social and economic benefits [3].

Telemedicine offers the opportunity to integrate communication between hospitals and territorial healthcare structures and services. Coordinated care and telehealth services have the potential to improve the quality of care, tailored to the needs, particularly of chronically ill patients [6].

Additionally, telehealth provides the opportunity to bring remote care to the child expanding school-based telehealth programs. These programs may offer an early intervention means for children with chronic illnesses, such as diabetes, epilepsy, asthma, and may address behavioral and educational issues [7].

The benefits of telemedicine to deliver pediatric services to medically underserved regions and low-income countries must be also considered [2].

Even though telemedicine is likely to be more of a mobile and home-based practice it is important to consider the design of clinic-based telemedicine facilities and to optimize dedicated space for telemedicine [4]. A perspective of a pediatric virtual hospital may represent a challenge in pediatrics, offering an innovative access to health care. Telemedicine services for pediatric care are comparable to or better than in-person services, offering an alternative to traditional patient visits [2].

Despite the difficulties to access, the costs to maintain telemedicine instruments and the insufficient legal regulations are still barriers to telemedicine use, the innovative technologies represent a future health challenge.

The Special Issue “Telemedicine and E-health: Innovations in Care for Children” focuses on the current state of knowledge and experience on telemedicine and e-health in pediatrics as an innovative approach to children’s care.

We invite experts from different disciplines, including pediatrics, medical informatics, exercise and sports sciences, intervention studies, risk and health impact assessment, and preventive medicine, to submit all types of manuscripts; papers on epidemiological aspects and socio-economic impact of telehealth will be also considered.

To communicate new experiences, future research and initiatives are useful to make know the potential of telemedicine as a strategic approach for child’s health and pediatric health policy.
